# Lipoprotein(a) in Children and Adolescents: Risk or Causal Factor for Cardiovascular Disease? A Narrative Review

**DOI:** 10.3390/ijms25168817

**Published:** 2024-08-13

**Authors:** Maria Elena Capra, Giacomo Biasucci, Giuseppe Banderali, Cristina Pederiva

**Affiliations:** 1 Pediatrics and Neonatology Unit, Centre for Pediatric Dyslipidemias, Guglielmo da Saliceto Hospital, 29121 Piacenza, Italy; m.capra@ausl.pc.it; 2 Department of Translational Medical and Surgical Sciences, University of Parma, 43126 Parma, Italy; 3 Department of Medicine and Surgery, University of Parma, 43126 Parma, Italy; 4 Pediatrics Unit, Clinical Service for Dyslipidemias, Study and Prevention of Atherosclerosis in Childhood, ASST-Santi Paolo e Carlo, 20142 Milan, Italy; giuseppe.banderali@asst-santipaolocarlo.it (G.B.); cristina.pederiva@asst-santipaolocarlo.it (C.P.)

**Keywords:** lipoprotein(a), cardiovascular risk, children, adolescents, atherosclerosis

## Abstract

The evaluation of serum Lp(a) values in childhood and adolescence has been widely debated, and in the last few years, many authors have tried to better define Lp(a) role in atherosclerosis pathogenesis, starting from childhood. In our narrative review, we have evaluated the main historical stages of Lp(a) studies in childhood, trying to focus on pathogenic mechanisms linked to elevated serum Lp(a) values, starting from ischemic stroke and vascular damage, and to its possible direct involvement in premature atherosclerosis from childhood onwards. Historic manuscripts on Lp(a) in pediatric patients have mainly focused on serum Lp(a) values and increased stroke risk. More recently, many studies have evaluated Lp(a) as a coronary vascular disease (CVD) risk factor starting from childhood, especially related to a positive family history of premature CVD. Finally, only a few studies evaluated the role of Lp(a) in premature atherosclerotic processes and endothelial and vascular damage in pediatric patients. Lastly, we have hypothesized a future perspective, with the hope that plasma Lp(a) levels will be treated with a tailored pharmacologic approach, and Lp(a) will become a precocious therapeutic target to control the atherosclerotic pathways from the first years of life.

## 1. Introduction

Lipoprotein(a) (Lp(a)) is a serum lipoprotein formed by the binding of a particle rich in Low-Density Lipoprotein Cholesterol (LDL-C) to one molecule of Apolipoprotein B100 and Apolipoprotein(a) [1]. Lp(a) is structurally similar to LDL-C, but the presence of apo(a) is the principal causative factor that makes LDL and Lp(a) different as far as density, electrophoretic mobility, and molecular weight are concerned [2,3]. Lp(a) was first described in 1963 by the geneticist Kare Berg, who identified in human serum an LDL cholesterol antigen, which he denominated apolipoprotein(a) [3]. Berg also evaluated this particle distribution in some familiar clusters; he identified its genetic transmission, and in 1974, he suggested the association between elevated Lp(a) plasma levels and coronary heart disease. In the 1980s, many researchers confirmed Lp(a) role as a CVD risk factor, and the LPA gene was first sequenced [4]. In the 2000s, thanks to Mendelian randomization studies in adult subjects, the causal association between high Lp(a) serum levels and cardiovascular diseases was clearly defined [5,6]. Clarke et al. evaluated a wide gene panel linked to cardiovascular disease, highlighting that “genetic variations” in the LPA gene were strongly associated with cardiovascular disease genetic risk [7]. In recent years, Lp(a) has been increasingly studied as a cardiovascular disease risk factor and causative atherosclerosis factor. In 2022, the European Atherosclerosis Society signed a consensus on Lp(a) role in atherosclerosis and cardiovascular disease, stating that Lp(a) should be evaluated at least once in adult subjects, and it should always be considered in the context of absolute global cardiovascular disease risk and, when altered, treated [8]. Most studies on Lp(a) deal with adult subjects. Attention and focus on the role of Lp(a) in children and adolescents has constantly risen in the past years, even if many aspects of this field are yet to be explored. Our manuscript aims to depict a historical overview of Lp(a) role in pediatric subjects and discuss its role in the atherosclerotic process and cardiovascular disease in children and adolescents.

## 2. Methods

The MEDLINE–PubMed database was searched to detect and select papers from 1980 to 2024. The query encompassed randomized placebo-controlled trials, controlled clinical trials, double-blind, randomized controlled studies, and systematic reviews. The following combinations of keywords were used: “lipoprotein(a)” AND “children” OR “adolescents” OR “pediatric” OR “childhood” OR “ developmental age” AND “atherosclerosis” OR “cardiovascular disease.” We also performed a manual search of the reference lists of the selected studies. The search was limited to English-language journals and full papers only.

## 3. Biochemical Structure and Metabolism of Lp(a)

Lp(a) is structurally similar to plasmin and plasminogen: in fact, Apo(a) contains a specific protein domain called “kringle,” which is composed of more than 80 amino acids [9]. Single nucleotide polymorphisms (SNPs) of the LPA gene are fundamental determinants in apo(a) heterogeneity, as they affect RNA splicing [10]. Apo(a) precise mechanism of action has been widely investigated, but it has not yet been fully clarified. Experimental studies demonstrate that apo(a) acts in a regulatory way, both in the inflammation process and in wound reparation, and has a modulatory role in the cholesterol efflux capacity of cells [2]. The apo(a) component of the Lp(a) particle is encoded by the LPA gene, which is placed on the long arm of chromosome 6 within 6q2.6–2.7 [11], and it has a more than 70% homology if compared to the plasminogen gene [12].

Serum Lp(a) levels are dependent on the rate of hepatic synthesis of apolipoprotein(a): apolipoprotein(a) metabolism is still under study, but evidence supports the hypothesis that apolipoprotein(a) binds extracellularly and covalently to apolipoprotein B100-containing lipoproteins, predominantly LDL [13]. Liver secretion rates are lower for large apolipoprotein(a) isoforms; therefore, the smallest isoforms are typically predominant in the circulation. Lipoprotein(a) catabolism is carried out by both liver and kidney, but these metabolic pathways do not seem to interfere with serum Lp(a) values [1].

## 4. The Mechanism through Which Lp(a) Mediates Cardiovascular Disease

Lp(a) plays its pro-atherogenic action when it passes from the blood vessels to the arterial wall. Its concentration in the arterial wall depends on various factors, such as arterial wall permeability, Lp(a) plasma concentration, and arterial blood pressure [14]. Lp(a) tends to remain extracellular because it interacts with the extracellular matrix [2]. Lp(a) mediates atherosclerotic cardiovascular disease in various ways. Apolipoprotein(a) is a carrier of the same atherogenic risk of LDL particles, but it can also increase the inflammation process through its oxidized phospholipids. Moreover, Lp(a) has an antifibrinolytic effect that exerts an inhibitory action on plasminogen activation, and it accumulates in arterial walls thanks to its lysine binding sites [15]. The main hypothesis is that Lp(a) contributes independently to atherosclerosis pathogenesis through a combination of pro-inflammatory, prothrombogenic, and antifibrinolytic factors [16].

## 5. Serum Lp(a) Concentration

The number of Kringle-IV2 (K-IV2) copies determines the heterogeneity in apo(a) and, consequently, Lp(a) size. Lp(a) size is a fundamental factor in controlling the circulatory levels of Lp(a) [17]. Large studies reveal that polymorphisms in the LPA gene, such as rs783147, rs3798220, and rs10455872, are robustly linked with atherosclerotic lesions, increased carotid intima-media thickness and altered endothelial function: LPA gene SNPs seem to play a direct promoting role in the determination of early atherosclerotic changes, even if more significant evidence is necessary to define this relation better [18]. However, serum Lp(a) concentrations are determined not only by genetic aspects but also by biochemical structure and ethnic and geographical factors [8]. Biochemical characteristics of Lp(a) may influence its plasma concentration; in particular, the Kringle-IV (K-IV) repeat polymorphism explains up to 70% of the Lp(a) concentration variability, and when the number of repetitions is below the threshold of 23, apolipoprotein(a) is present in smaller isoforms, and Lp(a) plasma concentration increases [19]. Ethnicity plays a fundamental role in serum Lp(a) level determination, as demonstrated in various studies conducted on adult subjects [5]. The variations in Lp(a) values in different ethnic groups are mainly due to LPA gene variants and the size of Lp(a) isoforms. In the Dallas Heart Study, 3481 serum samples were from subjects of three ethnic groups belonging to a sample of the Dallas population composed of 50% Black subjects [19]. The authors of this study highlighted that elevated levels of pro-inflammatory oxidized phospholipids, of which Lp(a) is the main carrier, represent a genetic predisposition to the development of an increase in oxidative stress and that the differences in apolipoprotein(a) isoforms account for some of the racial differences in Lp(a) seen in this population [20]. In the Atherosclerosis Risk in Communities Study (ARIC Study), serum Lp(a) concentration and its relationship with coronary heart disease and ischemic stroke were evaluated in a cohort of 9851 white and 3467 black adult subjects: the authors found that Lp(a) values were positively associated with coronary and heart events, with a similarly strong relationship in blacks compared with whites [21]. Recent data from the UK Biobank analyzed the relationship between Lp(a) serum levels and atherosclerotic cardiovascular disease in a cohort of 460,506 subjects. The authors highlighted that, in the analyzed cohort, serum Lp(a) concentration varies across racial subgroups: median value was 16 nmol/L in Chinese, 19 nmol/L in White, 31 nmol/L in South Asian, and 75 nmol/L in Black individuals. However, the risk gradient resulted in a linear distribution across all ethnic groups with a similar pattern [22]. The serum Lp(a) concentration may also be modulated by non-genetically determined factors, such as diet and lifestyle. In a recently published review, the effect of nutritional habits on Lp(a) concentration in adult subjects was evaluated [23]. The authors analyzed several clinical trials and highlighted that nutrition exerts a slight effect on Lp(a) plasma values, and often not in the same direction as LDL-C. Further studies are needed to define better nutrition’s influence on Lp(a) metabolism.

The Lp(a) concentration is also influenced by those hormones that affect lipoprotein metabolism, such as thyroid hormone and growth hormone [24,25]. During pregnancy, serum Lp(a) values are often influenced by hormone changes. Pregnancy is characterized by a hypofibrinolytic state, and high Lp(a) may enhance fibrinolysis and play an unfavorable role in pregnancy outcome. In a longitudinal study conducted on pregnant women, the authors concluded that there is a 2-fold Lp(a) increase during normal pregnancy, and this may influence fibrinolysis [26].

Chronic kidney disease can influence Lp(a) plasma values as well. In subjects with nephrotic syndrome, serum Lp(a) values increase up to 5-fold compared with unaffected subjects [27].

The result of inflammatory status on Lp(a) metabolism has been widely debated, but no agreement has been reached yet. Missala et al. analyzed the connection between Lp(a) and autoimmune disease in a systematic review, concluding that anti-Lp(a) antibodies may be present in some autoimmune conditions, thus provoking an increase in Lp(a) values and a consequent increase in CHD risk. In these subjects, therapeutic approaches, such as Lp(a)-apheresis and Cholesteryl ester transfer protein (CETP) inhibitors, are currently under investigation [28]. Mooser et al. analyzed Lp(a) concentration in nine intensive care unit subjects affected by sepsis and four subjects with extensive burns, concluding that Lp(a) acts as a negative acute-phase reactant during major inflammatory response [29].

## 6. Lp(a) in Children and Adolescents

The growing interest in Lp(a) in children and adolescents has developed along with Lp(a) characterization and genetic definition. Historic manuscripts on Lp(a) in pediatric patients have mainly focused on serum Lp(a) values and increased stroke risk. More recently, many studies have evaluated Lp(a) as a cardiovascular disease (CVD) risk factor starting from childhood, especially related to a positive family history of premature CVD. On the contrary, only a few studies evaluated Lp(a) role in premature atherosclerotic process and endothelial and vascular damage in pediatric patients. We will discuss this evidence in detail in the following paragraphs.

## 7. Lp(a) and Stroke

Many studies have reported a link between high serum Lp(a) concentrations and the occurrence of arterial ischemic stroke. In 1996, Nowak-Göttl et al. [30] analyzed a cohort of 14 children and considered Lp(a) values as an ischemic stroke risk factor. The same authors reported a correlation between elevated Lp(a) values and incidence of both arterial and venous thrombosis in a cohort of 72 pediatric subjects [31]. A few years later, they discussed the role of Lp(a) as an ischemic stroke risk factor in a cohort of pediatric subjects aged 6 to 16 years, concluding that Lp(a) values higher than 30 mg/dl are strongly predictive of ischemic stroke (OR 7.2, 95% CI 3.8–13.8) [32]. The same year, Peynet et al. reported that young adults who experienced ischemic stroke had elevated Lp(a) concentrations, but they did not find a relationship between stroke incidence and apo(a) dimensions [33]. In 2010, a systematic review and meta-analysis of observational studies confirmed Lp(a) role as a causative risk factor for ischemic stroke and venous thrombosis in newborns and children [34], whereas in a further study, the association between Lp(a) values and arterial stroke in pediatric subjects was consolidated [35]. This finding was consistent with the conclusions of a meta-analysis published the following year on Atherosclerosis: the authors highlighted Lp(a) role as an independent ischemic stroke risk factor, especially in young subjects [36]. In a systematic review and meta-analysis on hereditary thrombosis and ischemic stroke, Lp(a) was not enumerated among risk factors [37], whereas in the EAS Consensus published in 2022, Lp(a) is not considered a risk factor for venous thrombosis, but it is associated with ischemic stroke also in pediatric patients [8].

## 8. Lp(a) and Cardio-Vascular Disease in Family Members

In the Bogalusa Heart Study, one of the most important historical studies on atherosclerosis pathogenesis, Lp(a) has been described in relation to positive family history for myocardial infarction (MI) in a cohort of 2438 children aged 8–17 years. Patients who had relatives with MI had higher Lp(a) concentrations if compared to those with a negative family history of MI (22.4 vs. 17.1 mg/dL), and this correlation was stronger in those who had Lp(a) level above 25 mg/dL, especially in Caucasian subjects. In the same study, the authors reported that subjects belonging to black ethnicity had more elevated average Lp(a) values. The authors concluded that serum Lp(a) level evaluation is of utmost importance in coronary disease risk evaluation and stratification, starting from childhood [38]. Bailleul et al. analyzed a cohort of 499 children, describing Lp(a) as a fundamental risk factor in relation to a positive family history of CVD in grandparents, concluding that testing serum Lp(a) in children is very important to identify those at increased CVD risk [39]. Many studies published in recent years have involved larger cohorts of subjects and have confirmed Lp(a) role as a risk factor for premature atherosclerosis starting from childhood [39,40,41,42,43,44]. Guardamagna et al described a positive relationship between Lp(a) levels and the number of CVD events in the family tree [45], whereas Zawacky et al. identified the Lp(a) value as the best predictor of premature CVD in the relatives of children with elevated Lp(a) if compared to those with elevated LDL-C plasma values [46]. In a study published in 2022, analyzing a cohort of 700 pediatric subjects with FH from the LIPIGEN pediatric group, the relationship between elevated serum Lp(a) values in children and a positive family history of premature CVD was confirmed [47]. A recent study described that Lp(a) values in young subjects are related to the incidence of major CVD events in adult age [48].

## 9. Lp(a) and Vascular Damage in Pediatric Patients

Some studies have analyzed the relationship between serum Lp(a) levels and functional and structural vascular alterations starting from the first years of life, with contrasting findings [49,50,51]. Sorensen et al. evaluated the association between endothelium-mediated dilation and serum Lp(a) levels in a cohort of thirty children (age 7–17 years) with FH. They found pathological alterations of the endothelial function starting from childhood, and what is more, the higher the serum Lp(a) levels are, the worse these alterations are [49]. Endothelial vessel alterations have also been confirmed by Lapinleimu et al. and by Qayun et al. in further studies [44,52]. In the Young Finns Study, a milestone in the cardiovascular risk studies, Lp(a) values have been evaluated as an atherosclerosis risk factor in young subjects through epidemiological and Mendelian randomization studies without evidence of any direct correlation [18]. Similarly, in a recent Austrian study on 113 children and adolescents aged 1 to 18 years, a retrospective analysis evidenced no significant correlation between Intima-Media Thickness (IMT) z score and serum Lp(a) values [53]. On the contrary, other studies supported the correlation between Lp(a) values and vascular damage starting from childhood. Kosmeri et al. evaluated Lp(a) levels in a cohort of 100 children and adolescents (aged 7–16 years), and they found that elevated brachial artery FMD (flow-mediated dilation) was independently associated with high Lp(a) values starting from 10 years of age [54]. De Boer et al. recently described the association between Lp(a) values and intima-media thickness in children with FH (214 subjects aged 8–18 years), evaluated in a 20-year follow-up study, and they highlighted the importance of Lp(a) evaluation starting from childhood, to early and promptly detect those subjects at higher CHD risk [55].

## 10. Lp(a) Values in Children and Adolescents

In the Consensus Document published in 2022, the authors reported that the LPA gene completes its full expression by the age of 20 years, whereas serum Lp(a) levels reach a stable value by the age of five years [8]. One of the first studies published on this topic compared serum Lp(a) levels of 44-term newborns with those of healthy adult subjects, finding that Lp(a) values in newborns are considerably lower if compared with adult ones [56]. A few further studies have evaluated Lp(a) at birth, finding that it relates to gestational age [57] and ethnicity [58,59], thus confirming a skewed distribution of Lp(a) levels in pediatric patients, as already demonstrated in the adult ones [60]. Wood et al. evaluated serum Lp(a) values in the first months of life in a cohort of 220 newborns, highlighting a weak correlation with Lp(a) values at birth and a tendency toward increased values in the first months of life [61]. In the Special Turku Coronary Risk Factor Intervention Project (STRIP) Study, Lp(a) levels were evaluated in a cohort of young children (430 children aged from 6 to 36 months), with checks at 7, 18, 24, and 36 months. At 7 months, Lp(a) values were correlated with those found at 36 months, confirming the remarkable stability of Lp(a) plasma levels, especially for very low and very high values [62]. This trend has been also confirmed in more recent studies. In the COMPARE study, serum Lp(a) value was evaluated in a cohort of 450 newborns from the Copenhagen population. If cord blood values (venous sample) and those at 2 and 15 months are considered, Lp(a) levels increase with age. Lp(a) values in cord blood are consistent with those evaluated at birth, whereas the correlation is weaker if we consider samples taken at 2 and 15 months. This correlation becomes evident when Lp(a) values at birth are >90° centile. The authors concluded that serum Lp(a) values above 90° centile at birth can identify newborns at risk of developing high Lp(a) values in later years [63]. De Boer et al. report that the older the children, the higher the Lp(a) values in a cohort of FH pediatric patients, suggesting the need for further determinations of serum Lp(a) in adult life [64].

## 11. Treatment Options for Elevated Lp(a)

As we have discussed, Lp(a) plasma values are mainly determined by genetic factors and are only partially modifiable by environmental factors. Some authors have analyzed lifestyle influence on Lp(a) plasma levels, especially regarding nutritional modifications and physical activity improvement, confirming that plasma Lp(a) values are scarcely modifiable by these factors. Over the past decades, pharmacological treatment for elevated Lp(a) plasma values has been widely debated [65]. At present, Lipoprotein apheresis (LA) is the only proven and effective therapy to lower Lp(a) serum levels in adult subjects. LA has proved to be very useful in lowering Lp(a) levels and may be clinically beneficial in high-risk subjects. LA is a time-consuming and invasive therapy; therefore, it is not feasible for pediatric patients with isolated Lp(a) and elevated levels [66,67].

In the past decades, niacin has been the first treatment for adult subjects with elevated Lp(a) plasma levels. Niacin is a B-complex vitamin related to a moderate decrease in Lp(a) plasma levels of up to 31% of basal levels [68]. The precise mechanisms through which niacin can lower Lp(a) values are yet not completely understood, but some studies state that niacin lowers both apo(a) and apo B production rates [68]. Niacin is often stopped because of adverse events such as hyperglycemia, flushing, and gastrointestinal symptoms [69], and its cardiovascular benefits are not clear; therefore, in Europe, niacin is indicated only for subjects with hypertriglyceridemia. Considering niacin’s unclear therapeutic effect and its low tolerability, it is not considered a valid option for adult or pediatric subjects with elevated Lp(a).

Statins are HMG-CoA reductase inhibitors used to lower total and LDL cholesterol plasma values. They can be used in children with FH starting at 8 years of age [8,70]. Lp(a) and LDL-cholesterol are structurally similar, so drugs used to lower LDL-cholesterol may also affect Lp(a) plasma values. Unfortunately, this hypothesis has not been confirmed by clinical trials, as reported in a recent meta-analysis conducted by de Boer et al. [71]. The authors conducted a systematic review and meta-analysis of randomized trials with a statin and placebo arm and concluded that statin therapy does not have a statistically significant lowering effect on Lp(a) plasma values.

Ezetimibe is an inhibitor of cholesterol intestinal absorption through a selective binding to the cholesterol transporter Niemann-Pick C1-Like 1 (NPC1L1). Awad et al. [72] evaluated the ezetimibe effect on Lp(a) plasma levels in a metanalysis, highlighting a tiny but significant lowering action on Lp(a) plasma values. On the contrary, Sahebkar et al. conducted a systematic review that did not confirm the ezetimibe effect on Lp(a) plasma values [73].

PCSK9 inhibitors can lower Lp(a) plasma values up to 20–30% of basal values in adult subjects [74,75,76]. As we have pointed out before, statin trials that documented a statin-induced increase in LDL-R did not evidence a significative Lp(a) serum level reduction, suggesting that LDL-R exerts little effect on Lp(a) catabolism. PCSK9 inhibitors determine an overexpression of LDL-R; however, they can have a greater effect on Lp(a) serum levels, as well as in subjects with HoFH, who have very low residual LDLR activity. These findings led to the hypothesis that PCSK9 inhibitors can act on Lp(a) metabolism independently from LDL-R, and some researchers confirmed that PCSK9 inhibitors can also modulate Lp(a) synthesis and not only its catabolism [77,78,79]. Some studies have evaluated the effect of PCSK9 inhibitors on Lp(a) in pediatric patients as a secondary or tertiary outcome. In the ODISSEY KIDS study, the effect of alirocumab was evaluated in a cohort of pediatric patients with FH, and the authors reported a main reduction of 2 to 15% of Lp(a) plasma values [65]. Similar results have also been reported with evolocumab treatment in pediatric patients with HeFH, with a mean reduction of 10% of Lp(a) plasma values. PCSK9 inhibitors have recently been approved in pediatric patients with HoFH, and we can hypothesize that they will be used to reach optimal LDL-C levels in subjects with FH and elevated Lp(a) plasma values, even if at present, there are no specific studies and no clear evidence. PCSK9 inhibitors could be used to lower the overall cardiovascular risk in pediatric subjects with elevated Lp(a) plasma levels, but they are not indicated as a target therapy to lower Lp(a) [79].

Mipomersen is an antisense oligonucleotide (ASO) that inhibits apoB protein translation, thus reducing apoB production and, as a consequence, lowering the liver synthesis of apoB-containing lipoproteins such as LDL-C and Lp(a) [69]. Mipomersen results in a lowering of 17 to 27% of Lp(a) plasma values, but adverse events such as flu-like symptoms and injection site reactions have been described; therefore, its use is authorized in the US for subjects with homozygous FH, and it is not recommended in Europe yet [65]. Raal et al. evaluated the effects of mipomersen on the lipid profile of a small cohort of pediatric patients with homozygous FH, and they found that mipomersen effectively reduced LDL-C levels but had a very modest effect on Lp(a) [80].

Cholesteryl ester transfer protein (CETP) is a glycoprotein found in plasma whose action is transporting cholesterol esters from high-density lipoprotein cholesterol (HDL-C) to ApoB-containing lipoproteins: when CETP is inhibited, so is these transfers’ rate, thus increasing HDL-C and lowering VLDLs and LDLs and slightly reduced Lp(a) levels [81]. Anacetrapib was reported to lower Lp(a) serum levels up to 40% of basal values, whereas obicetrapib has been reported to reach up to 55% of reduction. CETP inhibitors’ effect on Lp(a) metabolism is not yet fully understood, but some kinetic studies on anacetrapib reported a reduction in apolipoprotein(a) production related to drug use [81]. CETP inhibitors’ effects on Lp(a) plasma levels are not homogeneous and yet partially unclear.

**Figure 1:**
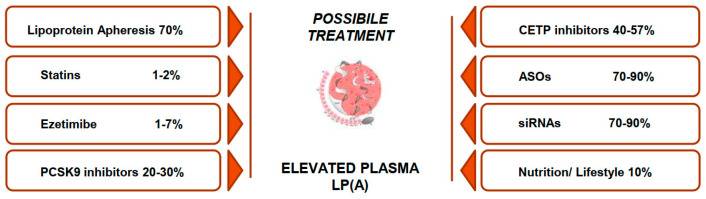
Treatment options for elevated plasma Lp(a) and average percentage of Lp(a) serum level reduction.

In the past few years, new therapies have been elaborated to prevent the assembling of the Lp(a) particle rather than increasing its catabolism: ASOs and siRNAs are the two main therapeutic options elaborated in this sense, even if they have not been tested in pediatric patients yet and they are administered through injection, so they are usually not well tolerated by pediatric patients [82]. Shortly, children and adolescents with both FH and elevated Lp(a) plasma levels and children with stroke and elevated Lp(a) plasma levels as the only possible explanation of the stroke could be two target populations for ASOs and siRNA [65]. As long as no therapeutic options are approved for children with high Lp(a) so far, prompt and optimization of other risk factors for CVD in pediatric subjects with elevated Lp(a) plasma levels should be a milestone in patients’ management [65]. Treatment options for elevated plasma Lp(a) levels are summarized in Figure 1.

## 12. Lp(a): Present and Future Perspectives

In recent years, thanks to the development of new therapeutic options [83,84,85], interest in Lp(a) has consistently grown, and many projects have confirmed Lp(a) role as a CHD and premature atherosclerosis risk factor. Sagris et al. evaluated characteristics and differences in myocardial infarction in young and old subjects, and elevated serum Lp(a) values were highlighted as causative risk factors, especially in young patients [86]. Olmastroni et al. smartly highlighted that Lp(a) genotype influences clinical FH diagnosis and remarked on the importance of Lp(a) values evaluation in the context of a correct FH diagnosis [87]. Averna et al. [88] briefly commented on a study published by De Boer et al., stating that Lp(a) can be considered a genetic cause of FH in children and adolescents [89]. De Boer et al. recently described Lp(a) values distribution in children with suspected FH: the authors reported higher Lp(a) values in subjects with a clinical diagnosis of FH, supporting Lp(a) role as a causative factor in subjects with clinical FH, when a genetic diagnosis of FH has not been reached [89]. Reeskamp et al. recently analyzed serum Lp(a) values in a large population (data from UK Biobank studies and random pair of unrelated individuals) of subjects with elevated Lp(a) values, relating their values with those of their first and second-degree relatives. They found a strong correlation between Lp(a) values and elevated values both in first and second-degree relatives [90]. The importance of Lp(a) values evaluation has been confirmed by numerous studies [16], both in an FH cascade screening context [91] and in an independent cascade screening, conducted to early detect subjects with elevated serum Lp(a) values [92], or even as a pediatric evaluation of a future risk [48]. After the EAS Consensus publication [8], serum Lp(a) values evaluation has been highlighted both as a CVD risk factor and as a CVD causative risk factor [93], and all the main consensus documents on cardiovascular risk in children and adolescents have agreed on this recommendation [94,95].

Soon, new specific therapeutic options to reduce serum Lp(a) levels will be available, and we will be able to make a further contribution to atherosclerosis prevention and early treatment [83,84,85].

## Data Availability

All data presented in this article are sourced from previously published articles referenced herein. Specific references to the sources of these data can be found in the reference list. No new data were generated or analyzed for this study.

## Abbreviations

Apo(a)	Apolipoprotein a
ARIC	Atherosclerosis Risk in Communities Study
CETP	Cholesteryl ester transfer protein
CHD	Coronary Heart Disease
CVD	Cardiovascular Disease
EAS	European Atherosclerosis Society
FH	Familial Hypercholesterolemia
FMD	Flow Mediated Dilation
HeFH	Heterozygous Familial Hypercholesterolemia
HoFH	Homozygous Familial Hypercholesterolemia
IMT	Intima-Media Thickness
KIV	Kringle IV
LDL	Low-Density Lipoprotein
Lp(a)	Lipoprotein a
PCSK9	Proprotein Convertase Subtilisin/Kexin type 9
SNPs	Single nucleotide polymorphisms
STRIP	Special Turku Coronary Risk Factor Intervention Project
UK	United Kingdom
